# Endoscopic Evaluation of Peptic Ulcer Disease During Ramadan Fasting

**DOI:** 10.1155/DTE.2.219

**Published:** 1996

**Authors:** G. M. Malik, M. Mubarik, G. Jeelani, H. Tajamul, S. A. Kadla, B. A. Lone, M. D. Khan

**Affiliations:** 1 Department of Medicine S.M.H.S. Hospital Srinagar Affliated to Government Medical College Srinagar Kashmir India; 2 c/o Qamerwari SK Coloney (Sec 1A) p/o Karan Nagar Srinagar Kashmir 190009 India

## Abstract

The effects of fasting on peptic ulcer disease were evaluated in a prospective study, involving 23 fasting patients who underwent endoscopy before and after Ramadan. Eighteen patients took an H_2_-blocker (ranitidine, 150 mg twice daily) regularly at “Suhur” and “Iftar” as prescribed, while 5 were drug defaulters. On the first endoscopy the diagnosis in 4 patients was active acute duodenal ulcer (AADU), in 8 patients was active chronic duodenal ulcer (ACDU) in 8 patients was healed duodenal ulcer (HDU), in 2 patients was erosive duodenitis (ED), and in 1 patient was chronic gastric ulcer (CGU). All of the patients with AADU showed signs of healing on repeat endoscopy. None of the ACDU patients showed signs of healing on repeat endoscopy. Instead, 7 patients in this group bled during fasting. All of the 5 drug defaulters belonged to the ACDU group. One patient in the HDU group had developed an active ulcer near the previous scar, as which was seen on repeat endoscopy. The 2 patients with ED showed signs of healing, while the only patient with CGU had bled from the same ulcer as seen on repeat endoscopy. The results were compared with those of 15 nonfasting control subjects (6 patients with ACDU, 3 with HDU, and 6 with ED as diagnosed on the first endoscopy), who took an H_2_-blocker regularly. The repeat endoscopy did not show any change in these patients. In conclusion, we inferred that Ramadan fasting may prove hazardous in patients with peptic ulcer disease in general and with active chronic ulcers in particular, although the fact that only 23 patients volunteered for this study, of whom 5 were drug defaulters, is a limitation.


*Diagnostic and Therapeutic Endoscopy*, 1996, Vol. 2, pp. 219–221
Reprints available directly from the publisher
Photocopying permitted by license only

© 1996 OPA (Overseas Publishers Association)
Amsterdam B. V. Published in The Netherlands
by Harwood Academic Publishers GmbH
Printed in Singapore

### Endoscopic Evaluation of Peptic Ulcer Disease During Ramadan Fasting: A Preliminary Study

G. M. MALIK, M. MUBARIK, G. JEELANI, H. TAJAMUL, S. A. KADLA, B. A. LONE, and M. D. KHAN

*Department of Medicine, S.M.H.S. Hospital Srinagar Affliated to Government Medical College Srinagar, Kashmir, India*

*(Received January 20, 1995; in final form August 7, 1995)*

The effects of fasting on peptic ulcer disease were evaluated in a prospective study, involving 23 fasting patients who underwent endoscopy before and after Ramadan. Eighteen patients took an $H_2$-blocker (ranitidine, 150 mg twice daily) regularly at "Suhur" and "Iftar" as prescribed, while 5 were drug defaulters. On the first endoscopy the diagnosis in 4 patients was active acute duodenal ulcer (AADU), in 8 patients was active chronic duodenal ulcer (ACDU) in 8 patients was healed duodenal ulcer (HDU), in 2 patients was erosive duodenitis (ED), and in 1 patient was chronic gastric ulcer (CGU). All of the patients with AADU showed signs of healing on repeat endoscopy. None of the ACDU patients showed signs of healing on repeat endoscopy. Instead, 7 patients in this group bled during fasting. All of the 5 drug defaulters belonged to the ACDU group. One patient in the HDU group had developed an active ulcer near the previous scar, as which was seen on repeat endoscopy. The 2 patients with ED showed signs of healing, while the only patient with CGU had bled from the same ulcer as seen on repeat endoscopy. The results were compared with those of 15 nonfasting control subjects (6 patients with ACDU, 3 with HDU, and 6 with ED as diagnosed on the first endoscopy), who took an $H_2$-blocker regularly. The repeat endoscopy did not show any change in these patients. In conclusion, we inferred that Ramadan fasting may prove hazardous in patients with peptic ulcer disease in general and with active chronic ulcers in particular, although the fact that only 23 patients volunteered for this study, of whom 5 were drug defaulters, is a limitation.

**KEY WORDS:** Ramadan, fasting, ulcer, duodenal, gastric, endoscopy

### INTRODUCTION

Ramadan is the ninth lunar month of the Muslim year during which it is obligatory for all healthy adult Muslims to observe a fast from dawn to sunset (1). The sick, those travelling, and pregnant, lactating, and menstruating women are permitted to postpone their observation of Ramadan fasting till some other suitable time. People may eat or drink at night but not during the day. After sunset they consume a large meal called "Iftar" and the last meal they consume is "Suhur" just before "Fajr" (dawn) prayers. The food during Ramadan is basically similar to the ordinary food consumed during the other 11 months, except that a large quantity is taken at Iftar and Suhur instead of several meals during the day(2).

The physiological changes during Ramadan are not fully known. Doctors have been concerned about the effects of Ramadan fasting on acid peptic disease and the effects of having a heavy meal following several hours of fasting. Should a patient with peptic ulcer disease observe a fast? It is a difficult question to answer. Since no such study has been carried out so far, we conducted a prospective study in patients with endoscopically proved peptic ulcer disease, who voluntarily observed the Ramadan fast. The patients were taking $H_2$-blockers and underwent repeat endoscopy after Ramadan to see the effects of fasting on their ulcer disease. The results were compared with 15 nonfasting control subjects.

### MATERIALS AND METHODS

The study was conducted in 23 patients with endoscopically proved peptic ulcer disease, who volunteered to observe the fast of Ramadan (1414 Hijri/February to March 1994), with 15 nonfasting patients acting as control subjects. $H_2$-blockers were prescribed for both the fasting and the nonfasting patients, to be taken at Suhur and Iftar. The endoscopy was done 7 days before and 7 days after the

Address for correspondence: Dr. G. M. Malik, c/o Qamerwari SK Coloney (Sec 1A), p/o Karan Nagar 190009 Srinagar, Kashmir, India.

fasting month in the Gastroenterology Department of S.M.H.S. Hospital, Srinagar. Of the 23 fasting patients, 20 (87%) were men and 3 (13%) were women, while the control group comprised 9 (60%) men and 6 (40%) women. Both the study and the control groups were in the age group of 21 to 65 years.

Active acute ulcer was defined as an area of denuded epithelium (5 mm) with or without slough at the base. Active chronic ulcer was defined as an ulcer with or without slough at the base with scarring and deformity. Healed ulcer was inferred, if endoscopy revealed a scar with or without deformity. Peptic ulcer included duodenal ulcer and benign gastric ulcer. Any gastric ulcer detected on endoscopy was biopsied for histological examination (3). Erosions were defined as superficial, small, multiple denuded areas confined to the mucosa (4).

### RESULTS

### Study Group

Of the 23 fasting patients with endoscopically proved peptic ulcer disease before Ramadan, 18 patients took $H_2$-blockers regularly at Suhur and Iftar. Eight of the fasting patients had gastrointestinal bleeding, of whom 4 had both hematomesis and melena and 4 had melena alone. Four patients had the bleeding in the 5th week, 2 in the 3rd week, and 1 each in the 1st and the 4th weeks.

### Endoscopic Findings

Four patients who had an active duodenal ulcer before Ramadan showed a healed ulcer after Ramadan. All patients took $H_2$-blockers regularly.

Of the 8 patients who had an active chronic duodenal ulcer before Ramadan, 7 patients bled during Ramadan. Of these 6 showed signs of rebleeding from the same ulcer and in 1 no source was detected on repeat endoscopy. Five patients in this group had stopped the drugs and developed bleeding, while 3 continued the drugs regularly and 2 of these developed bleeding.

Of the 8 patients who had a healed duodenal ulcer on first endoscopy, 7 showed no change in the scar whereas 1 had developed an active ulcer near the previous scar as seen on repeat endoscopy. All took drugs during the fasting period.

The two patients having previous erosive duodenitis showed complete healing of the erosions at the end of Ramadan. Both had taken the drugs regularly throughout Ramadan.

One patient who had a chronic gastric ulcer on the high lesser curve on previous endoscopy showed no signs of healing and had bled during Ramadan fasting. He was taking the drugs regularly (Table 1). The repeat biopsy also showed a benign gastric ulcer. All fasting patients were consuming meals (rice, vegetable, and/or meat) and tea with bread at Suhur and Iftar.

### Control Group

The control group, who took $H_2$-blockers regularly comprised 6 patients with chronic duodenal ulcers, 3 with healed duodenal ulcers, and 6 with erosive duodenitis as diagnosed on the first endoscopy. No change was detected on repeat endoscopy in these patients (Table 1).

### DISCUSSION

During Ramadan some 100 million Muslims observe a fast from dawn to sunset (5). The period of fasting varies from 15 to 16 hours during the summer and 12 to 14 hours during the winter. The term "fasting" as applied during Ramadan is not fasting in the true sense, but rather it provides a unique model to study the effect of changing from eating several spaced meals a day to two large meals, one each at Iftar and Suhur. Previous work both in animals and humans has shown that the time distribution of food intake has a metabolic effect (1,6,7). There is a significant rise in serum cholesterol, uric acid, and thyroxine levels during fasting. No significant change has been observed in the serum levels of gastrin or insulin in the fasting state or 1/2 hour after consumption of food (2).

So far, no study has been conducted on the effects of Ramadan fasting on peptic ulcer disease. The present study was conducted in Kashmir, a Muslim-populated state of India, in which the point prevalence of peptic ulcers is 4.72% and the life-time prevalence is 11.22%. The duodenal to gastric ulcer ratio is 17.1:1, and both types of ulcers are more common in men (3). Most of the Muslims observe a fast even during an illness they think is not serious.

We observed that the patients with active acute duodenal ulcer showed signs of healing on treatment with $H_2$-blockers during Ramadan. Conversely, patients who had an active chronic duodenal ulcer showed no signs of healing, and 88% of these patients developed bleeding with 71% of these with bleeding being drug defaulters. The gastric ulcer also bled even after drug therapy and showed no signs of healing. It seems that chronic ulcers are difficult to heal during fasting. Patients who had a healed ulcer did not show much change at the end of fasting, but the erosions in the duodenum had healed on taking the drugs regularly.

The results of the study were affected because of variations in drug compliance by patients. Aslam and Healy (8,9)

**Table 1** Outcome in Patients Who Fasted During Ramadan and Comparison with Nonfasting Control Subjects

| *Lesion* | *No. of Patients* | *No. Who Took Treatment Regularly* | *Healed* | *Hemorrhage in* |
|---|---|---|---|---|
| Fasted | | | | |
| Active acute duodenal ulcer | 4 | 4 | 4 | |
| Active chronic duodenal ulcer | 8 | 3 | 0 | 7* |
| Healed duodenal ulcer | 8 | 8 | 7 | |
| Erosive duodenitis | 2 | 2 | 2 | |
| Chronic gastric ulcer | 1 | 1 | 0 | 1† |
| Total | 23 | 18 | 13 | 8 |
| Did not fast | | | | |
| Active chronic duodenal ulcer | 6 | 6 | 0 | |
| Healed duodenal ulcer | 3 | 3 | 3 | |
| Erosive duodenitis | 6 | 6 | 0 | |
| Total | 15 | 15 | 3 | 0 |

*All 5 drug defaulters bled as did 2 of 3 who complied.
†This patient was a complier who bled.

showed that of 44 Muslim patients attending an outpatient clinic, only 7 were prepared to take their medications in accordance with the prescriber's instructions during Ramadan. Our results indicate that fasting may prove hazardous in the patients with peptic ulcer disease in general and active chronic ulcers in particular, although our study had a limitation in that 5 patients (who all developed gastrointestinal bleeding) were drug defaulters. Thus, as in patients with hypertension, hyperuricemia, hyperlipidemia, and heart, liver, and kidney disease (1), Ramadan fasting may also produce some ill effects in patients with peptic ulcer disease. Such patients require both careful consultation with their doctors before continuing to observe fasting and supervision during the fasting period to avoid any crisis.

It must be pointed out that this study was performed on a small group of fasting patients during Ramadan. Large studies are, however, required to draw any final conclusions about the effects of Ramadan fasting on peptic ulcer disease.

### REFERENCES

1. El-Hazmi MAF, Al-Faleh FZ. Effects of Ramadan fasting on values of haematological and biochemical parameters. Saudi Med J 1987;8:171–176.
2. Suleiman FF, Murphy D, Salih Y, et al. Changes in certain blood constituents, during Ramadan. Am J Clin Nutr 1982;36:350–354.
3. Khuroo MS, Majajan R, Zargar SA, et al. Prevalence of peptic ulcer in India. An endoscopic and epidemiological study in urban Kashmir. Gut 1989;30:930–934.
4. Kimmey MB, Silverstein FE. Gastrointestinal endoscopy. In: Isselbacher, Brawnwald, Wilson, et al., eds. Harrison's principles of internal medicine, 13th ed. New York: McGraw-Hill, 1994: 1350–1355.
5. Aslam M, Wilson JV. Clinical problems during the fast of Ramadan. Lancet 1989;339:431–435.
6. Fabry P, Tapperman J. Meal frequency: A possible factor in human pathology, Am J Clin Nutr 1963;13:209–213.
7. Isabel MI, Ruth MF. Frequency and size of meals and serum lipids, nitrogen and mineral retention, fat digestibility and urinary thiamine and riboflavin in young women Am J Clin Nutr 1977;20:816–824.
8. Aslam M, Healy M. Drug regimes and fasting Muslim patients. Br Med J 1985;290:1746.
9. Aslam M, Healy M. Complaince and drug therapy in fasting Muslim patients J Clin Hosp Pharm 1986;11:1–5.

